# Longitudinal analysis of the ABCD® study

**DOI:** 10.1016/j.dcn.2025.101518

**Published:** 2025-02-08

**Authors:** Samuel W. Hawes, Andrew K. Littlefield, Daniel A. Lopez, Kenneth J. Sher, Erin L. Thompson, Raul Gonzalez, Laika Aguinaldo, Ashley R. Adams, Mohammadreza Bayat, Amy L. Byrd, Luis FS Castro-de-Araujo, Anthony Dick, Steven F. Heeringa, Christine M. Kaiver, Sarah M. Lehman, Lin Li, Janosch Linkersdörfer, Thomas J. Maullin-Sapey, Michael C. Neale, Thomas E. Nichols, Samantha Perlstein, Susan F. Tapert, Colin E. Vize, Margot Wagner, Rebecca Waller, Wesley K. Thompson

**Affiliations:** a Center for Children & Families, Florida International University, Miami, FL, USA; b Psychological Sciences, Texas Tech University, Lubbock, TX, USA; c Department of Psychiatry, Oregon Health & Science University, Portland, OR, USA; d Psychological Sciences, University of Missouri, Columbia, MO, USA; e Department of Psychiatry, University of California San Diego, San Diego, CA, USA; f Department of Psychiatry, University of Pittsburgh, Pittsburgh, PA, USA; g Virginia Institute for Psychiatric and Behavioral Genetics, Virginia Commonwealth University, Richmond, VA, USA; h Cognitive Neuorscience, Florida International University, Miami, FL, USA; i Institute for Social Research, University of Michigan, Ann Arbor, MI, USA; j Department of Radiology, University of California San Diego, San Diego, CA, USA; k Center for Human Development, University of California San Diego, San Diego, CA, USA; l School of Mathematics, University of Bristol, Bristol, United Kingdom; m Oxford Big Data Institute, University of Oxford, Oxford, United Kingdom; n Department of Psychology, University of Pennsylvania, Philadelphia, PA, USA; o Department of Psychology, University of Pittsburgh, Pittsburgh, PA, USA; p The Institute for Neural Computation, University of California San Diego, San Diego, CA, USA; q Center for Population Neuroscience and Genetics, Laureate Institute for Brain Research, Tulsa, OK, USA

**Keywords:** Longitudinal (nalysis, ABCD (tudy, Development

## Abstract

The Adolescent Brain Cognitive Development® (ABCD) Study provides a unique opportunity to investigate developmental processes in a large, diverse cohort of youths, aged approximately 9–10 at baseline and assessed annually for 10 years. Given the size and complexity of the ABCD Study, researchers analyzing its data will encounter a myriad of methodological and analytical considerations. This review provides an examination of key concepts and techniques related to longitudinal analyses of the ABCD Study data, including: (1) characterization of the factors associated with variation in developmental trajectories; (2) assessment of how level and timing of exposures may impact subsequent development; (3) quantification of how variation in developmental domains may be associated with outcomes, including mediation models and reciprocal relationships. We emphasize the importance of selecting appropriate statistical models to address these research questions. By presenting the advantages and potential challenges of longitudinal analyses in the ABCD Study, this review seeks to equip researchers with foundational knowledge and tools to make informed decisions as they navigate and effectively analyze and interpret the multi-dimensional longitudinal data currently available.

## 1 Introduction

The Adolescent Brain Cognitive Development (ABCD) Study® is the largest longitudinal investigation of neurodevelopment and child health in the United States. Conceived and initiated by the National Institutes of Health (NIH), this landmark prospective longitudinal study aims to transform our understanding of the genetic and environmental factors impacting neurodevelopment and their roles in behavioral and health outcomes across ten years of adolescence Volkow et al. (2018). At its heart, the study is designed to chart the course of human development across multiple interacting domains from late childhood to early adulthood and to identify factors that lead to both positive and negative outcomes. Central to achieving these goals is the commitment of the ABCD Study and its NIH funders to an open science framework, intended to facilitate sharing of data and analytical methods by espousing practices that increase access, integrity, and reproducibility of scientific research. In this context, the ABCD Study is a collaboration with the broader research community.

The size and scope of the ABCD Study data allow the research community to perform a large variety of developmental analyses of both substantive and methodological interest, presenting a unique opportunity to significantly advance our understanding of how a multitude of biopsychosocial processes unfold across critical periods of development. In this paper, we describe models and methods for longitudinal analysis of ABCD Study data that can address these fundamental scientific aims, including: (1) characterization of the genetic and environmental factors associated with variation in developmental trajectories; (2) assessment of how the level and timing of exposures may impact subsequent neurodevelopment; (3) quantification of how variation in developmental domains may be associated with outcomes, including mediation models and reciprocal relationships. We instantiate these longitudinal analyses in worked examples which are accessible along with accompanying scripts and code, at the project’s website, longitudinal.dev (https://longitudinal.dev/).

### 1.1 The ABCD study data

The ABCD Study enrolled a cohort of n = 11,880 participants born between 2006 and 2008 and aged approximately 9–10 years at baseline, each with a parent/guardian. The study sample was recruited from households in defined catchment areas for each of the 21 (originally 22) study sites across the United States. Information regarding funding agencies, recruitment sites, investigators, and project organizations can be obtained at https://abcdstudy.org. The ABCD Study design is described in more detail in Garavan et al. (2018) and Dick et al. (2021).

The ABCD Study is currently collecting longitudinal data on a rich variety of outcomes that will enable the construction of complex statistical models, potentially incorporating factors from many domains. Each new wave of data collection provides another building block for characterizing developmental trajectories and implementing longitudinal analyses that allow researchers to characterize normative development, to identify variables that presage deviations from normative development, and to assess a range of variables associated with biopsychosocial outcomes of interest. These data include: (1) a neurocognitive battery (Luciana et al., 2018, Thompson et al., 2019); (2) mental and physical health assessments (Barch et al., 2018); (3) measures of culture and environment (Gonzalez et al., 2021, Zucker et al., 2018); (4) substance use (Lisdahl et al., 2021); (5) gender identity and sexual health (Potter et al., 2022); (6) biospecimens (Uban et al., 2018); (7) structural and functional brain imaging (Casey et al., 2018, Hagler et al., 2019, Palmer et al., 2022); (8) geolocation-based environmental exposure data (Fan et al., 2021); (9) wearables and mobile technology (Bagot et al., 2018); and (10) whole-genome genotyping (Loughnan et al., 2020). Many of these measures are collected at in-person annual visits, with brain imaging collected at baseline and every other year going forward. A limited number of assessments are collected in semi-annual brief telephone or online assessments.

Data are publicly released approximately annually. The study’s earliest data releases consisted primarily of one or two visits per participant. However, the most recent public release as of the writing of this paper (Release 6.0) contains data collected across six annual visits, including three brain imaging assessments (baseline, year 2 follow-up, and year 4 follow-up visits). Hence, starting with Release 6.0, it is feasible for researchers to begin focusing on the characterization of neurodevelopmental and other trajectories.

**Table:** 

Organization and Aims

• Part I. Introduction o The ABCD Study® • Part II. Developmental Research o Fundamental Concepts • Part III. Longitudinal Analysis o Methods & Analysis • Part IV. Online materials o Linked open-source resources

## 2 Developmental research

### 2.1 Basic concepts and considerations

There are several important concepts to consider when conducting longitudinal analyses in a developmental context. These include different ways of thinking about the developmental course, whether certain periods of development are relatively sensitive or insensitive to various types of insults or stressors, whether some time periods or situations inhibit the expression of individual differences due to extreme environmental pressures, and whether the same behavior manifested at different times represents the same or different phenomena.

Moreover, in the case of developmentally-focused longitudinal research, each new measurement occasion not only provides a more extended portrait of the child’s life course but also brings with it greater methodological opportunities to make use of statistical models that distinguish within- from between-person effects and that loosen statistical constraints necessitated by fewer measurement occasions. For example, collecting two or more within-person observations on the same construct at different times enables estimation of individual rates of change (slopes) where more observations allow for more precise estimates of individual slopes (random slopes), as well as characterization of non-linear development. Rate of change or other trajectory characteristics may be more informative about individuals than the simple snapshots of level differences that cross-sectional data are limited to informing about. Cross-sectional age-related differences across individuals are poor substitutes for longitudinal trajectory estimates, except under highly restrictive assumptions (e.g., parallel trajectories and lack of age, cohort and experience effects; Thompson et al., 2011). Appreciation of these and other issues can help to guide the analysis and interpretation of data and aid translation to clinical and public health applications.

#### 2.1.1 Vulnerable periods

Adolescent development progresses normatively from less mature to more mature levels of functioning. However, unique epochs and experiences can alter the course of this idealized form of development. Consider research that shows cannabis use during adolescence is associated with later psychosis to a greater degree than cannabis use initiated later in development (Arseneault et al., 2002, Bechtold et al., 2016, Hasan et al., 2020, Semple et al., 2005). Similarly, rodent brains are especially sensitive to the neurotoxic effects of alcohol on brain structure and learning early in development, corresponding to early adolescence in humans (Spear, 2016, Crews et al., 2000, Ji et al., 2018). In another example, longitudinal data from the National Consortium on Alcohol and Neurodevelopment in Adolescence (NCANDA) show that binge drinking is associated more strongly with decrements in gray matter volume early in adolescence compared to later (Infante et al., 2022). These examples highlight the importance of considering the role of vulnerable periods – e.g., temporal windows of rapid brain development or remodeling during which the effects of environmental stimuli on the developing brain may be particularly pronounced– when trying to establish an accurate understanding of the association between exposures and outcomes.

#### 2.1.2 Developmental disturbances

Whereas vulnerable periods heighten neurobiological susceptibility to environmental influences, at other times, environmental exposures will tend to suppress stability and disrupt the orderly stochastic process of normative development (Schulenberg et al., 2019). This situation reflects a developmental disturbance in that the normal course of development is “altered” for a time by some time-limited process. In such cases, we might find that prediction of behavior in the period of the disturbance is reduced and/or, similarly, the behavior exhibited during the disturbance might have less predictive power with respect to distal outcomes compared to the behavior exhibited before and following the disrupted period. That is, once the environmental pressures are removed (or the individual is removed from the environment), patterns of individual differences (and autoregressive effects) recover to levels similar to those prior to entering the environment.

#### 2.1.3 Developmental snares and cascade effects

Normative development can also be upended by experiences (e.g., drug use) that, through various mechanisms, disrupt the normal flow of development wherein each stage establishes a platform for the next. For instance, substance use could lead to association with deviant peers, precluding opportunities for learning various adaptive skills and prosocial behaviors, in effect creating a “snare” that delays psychosocial development, such as maturing out of adolescent antisocial behavior (Moffitt, 2015). Relatedly, the consequences of these types of events can cascade (e.g., school dropout, involvement in the criminal justice system) so that the effects of the snare are amplified (e.g., Masten et al., 2005; Rogosch et al., 2010). Although conceptually distinct from vulnerable periods, both types of developmental considerations highlight the importance of viewing behavior in the context of development and attempting to determine how various developmental pathways unfold. Longitudinal data are crucial in this context to assess individual levels of development prior to and following onset of experiences or other environmental factors (e.g., the ABCD Study collected data starting at approximately ages 9–10 and hence before the onset of substance use for the vast majority of participants).

#### 2.1.4 Mediational Processes

Questions regarding the biological mechanisms whereby exposures impact outcomes can often be framed in terms of mediation analyses (MacKinnon et al., 2007, VanderWeele, 2016). Mediation analyses can be implemented using the causal steps approach (Baron and Kenny, 1986) and structural equation models (SEM) (Preacher et al., 2011). More recently, mediation models have been adapted for longitudinal exposures, mediators, and/or outcomes (Bind et al., 2016; VanderWeele & Tchetgen 2017). All of these modeling approaches decompose the total effects of an exposure on an outcome into direct and indirect effects, where indirect effects of an exposure flow through its impact on a mediating process. VanderWeele and Tchetgen (2017) detail conditions under which the direct and indirect causal effects can be in a longitudinal setting. An important example of mediational analyses in the ABCD Study is the impact of exposures on behavioral outcomes (e.g., neurocognition, mental health, substance use) via their impact on the brain, as quantified by imaging-derived phenotypes (IDPs). Methods for mediational analyses using multi-dimensional IDPs have been developed and applied to functional MRI data (e.g., Lindquist, 2012; Zhao et al., 2018).

## 3 Longitudinal data

### 3.1 Considerations and challenges

The hallmark characteristic of longitudinal data analysis (LDA) is the administration of repeated measurements of the same constructs on assessment targets (e.g., individuals, families) across time. The primary rationale for collecting longitudinal data is to assess within-person change over time, allowing researchers to estimate individual developmental trajectories and the genetic and person-level factors that may impact these trajectories. Administering repeated measurements more frequently or over longer periods enables researchers to ask more nuanced questions and to make stronger inferences.

#### 3.1.1 Two time points versus three or more

Although the clear leap from cross-sectional to the realm of longitudinal data involves going from one assessment to two or more assessments, there are also notable distinctions in designs based on two-assessment points versus three or more measurement occasions. Just as cross-sectional data can be informative in some situations, two waves of data can be beneficial in contexts such as when an exposure is involved (e.g., pre/post tests), or if the central goal is prediction (e.g., trying to predict scores on Variable A at time T as a function of prior scores on Variable A and Variable B at time T-1). At the same time, analyses of data based on two assessments are inherently limited on multiple fronts. As Rogosa et al. (1982) noted over forty years ago, “Two waves of data are better than one, but maybe not much better” (p. 744).

These sentiments are reflected in more contemporary recommendations regarding best-practice guidelines for prospective data, which increasingly emphasize the benefits of additional measurement occasions for trajectory estimation, model identification and accurate parameter inferences. This is also consistent with recommendations that developmental studies include three or more assessment points, given it is impossible for data based on two-time points to determine the shape of development (given that linear change is the only estimable form for two assessment waves; (see Duncan and Duncan, 2009). Research designs that include three (but preferably more) time points allow for non-linear trajectory estimation and increasingly nuanced analyses that more adequately tease apart sources of variation and covariation among the repeated assessments (King et al., 2018)– a key aspect of developmental research.

To illustrate, developmental theories are useful for understanding patterns of within-individual change over time (discussed in further detail, below); however, two data points provide meager information on change at the person level. This point is further underscored in a recent review of statistical models commonly touted as distinguishing within-individual vs between-individual sources of variance in which the study authors concluded “… researchers are limited when attempting to differentiate these sources of variation in psychological phenomenon when using two waves of data” and perhaps more concerning, “…the models discussed here do not offer a feasible way to overcome these inherent limitations” (Littlefield et al., 2021). It is important to note, however, that despite the current focus on two-wave designs versus three or more assessment waves, garnering three assessment points is not a panacea for longitudinal modeling. Indeed, several contemporary longitudinal models designed to isolate within-individual variability (e.g., the Latent Curve Model with Structured Residuals [LCM: SR]; Curran et al., 2014) require at least four assessments to parameterize fully and, more generally, increasingly accurate and nuanced parameter estimates are obtained as more assessment occasions are used (Duncan and Duncan, 2009).

#### 3.1.2 Types of stability and change

If one were to try to sum up what developmental trajectories in a living organism are exactly, one could plausibly argue they are the patterns of stability and change in its phenotypes as the organism traverses the life course. Symbolically, developmental trajectories can be expressed as fi(t), a possibly multivariate function of time t, specific to the ith individual and typically taking values in the real numbers for continuous phenotypes and the integers for discrete phenotypes. Ideally, t is a biologically meaningful temporal index (e.g., calendar age) as opposed to an exogenous progression of events (e.g., study visit number). Properties of interest might include rate of change over time, degree of smoothness (e.g., continuously differentiable), shape (e.g., polynomial or asymptotic behavior), how and how much f(t) differs across individuals, and what factors predict either within-individual variation (at different times) or between-individual variation (either overall or at specific times).

**Fig. 1:**
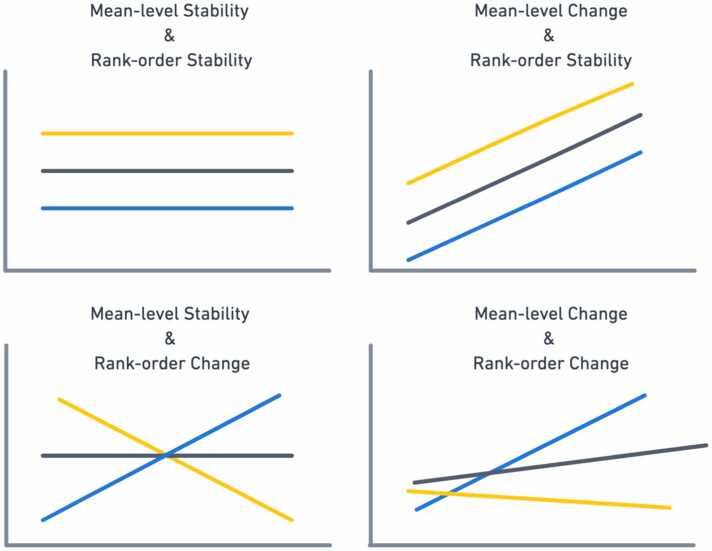
Types of Stability and Change.

There are a few different ways to think about patterns of stability and change (see Fig. 1). Consider measuring school disengagement at the start of middle school and the end of middle school. A common first step may be to compare sixth graders’ average disengagement values and eighth graders’ disengagement values. This comparison of the average scores for the same group of individuals at multiple time points is referred to as “mean-level”, as it provides information about change over time (or lack thereof) for an outcome of interest aggregated across members of a group. In contrast, “between-individual” stability could be assessed, e.g., by calculating the Spearman correlation between the values obtained at different time points (e.g., ‘disengagement in sixth grade’ with ’disengagement in eighth grade). This analysis focuses on the degree to which individuals retain their relative placement in a group across time. Consider someone who reported the lowest frequencies of disengagement in 6th grade and may report significantly higher disengagement over middle school (i.e., exhibit high levels of change), but report the lowest frequencies of disengagement in eighth grade. That is, the individual is manifesting rank-order stability, even in the context of high mean-level change.

Both types of stability and change are important. Mean-level change in certain traits might help to explain why, in general, populations of individuals tend to be particularly vulnerable to the effects of environmental factors in specific age ranges; rank-order stability might help to quantify the extent to which certain characteristics of the individual are more or less trait-like compared to others. For example, in some areas of development, considerable mean-level change occurs over time (e.g., changes in Big 5 personality traits; Bleidorn et al., 2022), but exhibit relatively high rank-order stability, at least over shorter measurement intervals (Bleidorn et al., 2022, Roberts and DelVecchio, 2000, Roberts et al., 2006).

Despite the useful information afforded by examining mean-level and rank-order stability and change, these approaches are limited in that they provide little information about the overall patterns of within-individual change and, in turn, can result in fundamental misinterpretations about substantial or meaningful changes in an outcome of interest (Curran and Bauer, 2011). For example, questions related to the impact of early-onset substance use on brain development focus on changes within a given individual (i.e., intraindividual differences). The ABCD Study will provide researchers with over ten time points for certain constructs (e.g., substance use) across a ten-year period, allowing for a detailed study of some within-person processes.

#### 3.1.3 Use of appropriate longitudinal models

There is growing recognition that statistical models commonly applied to longitudinal data often fail to align with the developmental theory they are being used to assess (Curran and Bauer, 2011, Hoffman, 2015, Littlefield et al., 2021). First, developmental studies typically involve the use of prospective data to inform theories that are concerned with clear within-person processes (e.g., how phenotypes change or remain stable within individuals over time, (Curran and Bauer, 2011). Despite this, methods generally unsuited for disaggregating between- and within-person effects (e.g., cross-lagged panel models [CLPM]) remain common within various extant literatures. Fortunately, there exists a range of models that have been proposed to tease apart between- and within-person sources of variance across time (see Littlefield et al., 2021; Orth et al., 2021). Most of these contemporary alternatives incorporate time-specific latent variables to capture between-person sources of variance and model within-person deviations around an individual’s mean (or trait) level across time (e.g., random-intercept cross-lagged panel model [RI-CLPM]; Hamaker et al., 2015); latent curve models with structured residuals [LCM-SR]; Curran et al., 2014). It is important to note however that these models require multiple assessments waves (e.g., four or more to fully specify the LCM-SR) and additional expertise to overcome issues with model convergence, and appreciation of modeling assumptions when attempting to adjudicate among potential models in each research context (see Littlefield et al., 2021, for further discussion).

Second, many statistical models assume certain characteristics about the data to which they are being applied. Common assumptions of parametric statistical models (e.g., linear mixed-effects models) include normality and equality of variances. These assumptions should be carefully considered before finalizing analytical approaches, so that valid inferences can be made from the data, as violation of a model’s assumptions can substantively invalidate the interpretation of results. For example, longitudinal data can exhibit heterogeneous variability (i.e., the variance of the response changes over the duration of the study) that may need to be accounted for within a model. Another pertinent modeling assumption is whether trajectories are linear or non-linear. With two or three assessments per individual, usually only a linear model of within-person change is feasible.

As the study progresses and more time points are assessed, the potentially nonlinear aspects of trajectories can be assessed, for example using quadratic functions of time. Methods that make even fewer assumptions about trajectory shapes, such as nonparametric curve estimation at the mean (e.g., Generalized Additive Mixed Models [GAMMs]; Wood, 2017) and at the individual level (e.g., Functional Data Analysis [FDA]; Ramsay and Silverman, 2002) may also become useful. Note, baseline age in the ABCD Study ranges over two full years; for some outcomes it may be feasible to include a possibly nonlinear effect of baseline age along with a linear effect of within-person change in age even with only two or three assessment times (Thompson et al., 2013).

#### 3.1.4 Continuous and discrete outcomes

Repeated assessments within the ABCD Study can be based on continuous or discrete measures. Examples of discrete measures include repeated assessments of binary variables (e.g., past 12-month alcohol use disorder status measured across ten years), ordinal variables (e.g., caregiver-reported items measuring emotional and behavioral concerns via the Child Behavior Checklist including the categories of “Not True”, “Somewhat True”, and “Very True”), and count variables (e.g., number of cigarettes smoked per day). In many ways, the distributional assumptions of indicators used in longitudinal designs mirror the decision points and considerations when delineating across different types of discrete outcome variables, a topic that spans entire textbooks (e.g., see Lenz, 2016). For example, the Mplus manual (Muthén, 2017) includes examples of (a) censored and censored-inflated models, (b) linear growth models for binary or ordinal variables, (c) linear growth models for a count outcome assuming a Poisson model, and (d) linear growth models for a count outcome assuming a zero-inflated Poisson model. Beyond these highlighted examples, other distributions (e.g., negative binomial) can be assumed for the indicators when modeling longitudinal data (Ren et al., 2022). These models account for issues that may occur when working with discrete outcomes, including overdispersion, i.e., when the variance is higher than would be expected based on a given parametric distribution (see Lenz, 2016). Given the sheer breadth of issues relevant to determining adequate models for discrete outcomes, it is not uncommon for texts on LDA to only cover models and approaches that assume continuous variables (e.g., Little, 2013). However, some textbooks on categorical data analysis provide more detailed coverage of the myriad issues and modeling choices to consider when working with discrete outcomes: Lenz (2016), Chapter 11 for matched pair/two-assessment designs; Chapter 12 for marginal and transitional models for repeated designs, such as generalized estimating equations, and Chapter 13 for random effects models for discrete outcomes.

#### 3.1.5 Issues in attributing longitudinal change to development

Systematic changes over time in a variable of interest are not always attributable to development: various pitfalls with longitudinal data can complicate or even invalidate this conclusion. For example, if data missingness or participant dropout are related to the values of the outcome, changing sample composition as the study progresses can bias mean trajectory estimates (we describe this in more detail in Section 3.1.7 below). Another prerequisite for valid developmental interpretations of longitudinal data is to establish whether a construct is measured consistently over time (i.e., longitudinal measurement invariance; Liu et al., 2017; Van De Schoot et al., 2015; Willoughby et al., 2012). Establishing longitudinal measurement invariance makes it more likely that change over time for a given construct is attributable to individual development rather than merely a measurement artifact. For instance, one study using data from the ABCD Study (Brislin et al., 2023) found differential item functioning in two items from a brief delinquency measure, revealing significant bias in an arrest item across Black and White youth. More specifically, Black youth were more likely to report being arrested compared to White youth with similar levels of delinquency. Prevalence rates of delinquent behavior would have been severely biased if measurement invariance had not been tested. Alternatively, Vize et al. (2023) showed partially strong to strong evidence of longitudinal measurement invariance across broad externalizing dimensions in youth taking part in the ABCD Study, suggesting that changes observed over time in these constructs were not due to systematic measurement error, but likely reflect true developmental change.

In addition to measurement invariance, the reliability of measures over time is another crucial consideration in longitudinal research, particularly when interpreting changes in constructs like neuroimaging metrics. Reliability, or the consistency of a measurement across time, directly influences the validity of conclusions drawn from longitudinal data (Revelle and Condon, 2019). In the neuroimaging domain, test-retest reliability is especially pertinent, as it determines whether the observed changes in brain function or structure reflect true neural changes or are simply due to measurement error (Elliott et al., 2020). When reliability is low, the potential for measurement error increases, which can obscure true longitudinal effects or produce misleading results. For example, using the Intraclass Correlation Coefficient (ICC) as a reliability metric in neuroimaging studies can provide insights into the stability of fMRI measures across sessions, which is crucial for interpreting changes over time (Elliott et al., 2020). When modeling longitudinal data, reporting relevant reliability statistics is essential for enhancing the transparency and interpretability of the findings.

Observed patterns of growth and decline often differ between cross-sectional vs. longitudinal effects (Salthouse, 2014) where subjects gain increasing experience with the assessment with each successive measurement occasion. Such experience effects on cognitive functioning have been demonstrated in adolescent longitudinal samples similar to ABCD (Sullivan et al., 2017) and highlight the need to consider these effects and address them analytically. In the case of performance-based measures (e.g., matrix reasoning related to neurocognitive functioning; see Salthouse, 2014), this can be due to “learning” the task from previous test administrations (e.g., someone taking the test a second time performs better than they did the first time simply as a function of having taken it before). Even in the case of non-performance-based measures (e.g., levels of depression), where one cannot easily make the argument that one has acquired some task-specific skill through learning, it has been observed that respondents tend to endorse lower levels on subsequent assessments (e.g., Beck et al., 1961; French and Sutton, 2010) and this phenomenon has been well documented in research using structured diagnostic interviews (Robins, 1985). While it is typically assumed that individuals are rescinding or telling us less information on follow-up interviews, there is reason to suspect that in some cases the initial assessment may be artifactually elevated (see Shrout et al., 2018).

Some longitudinal studies, e.g., accelerated longitudinal designs (ALDs; Thompson et al., 2011) are especially well suited for discovering these effects and modeling them. While ABCD is not an ALD, the variability in age (and grade in school) at the time of baseline recruitment (approximately 9–10 years old) allows some measures, collected every year, to be conceptualized as an ALD (e.g., substance use; prosocial behavior; family conflict; screen time). It is also possible that in later waves, analyses will allow for disaggregating the confounded effects of age and the number of prior assessments. However, ABCD is fundamentally a single-cohort, longitudinal design, wherein number of prior assessments and age are mostly confounded, and for, perhaps, most analyses, the possible influence of experience effects needs to be kept in mind.

#### 3.1.6 Modeling covariance

A central issue for repeated measurements on an individual is how to account for the correlated nature of the data. Lack of independence of residuals across time occurs for longitudinal data with repeated assessments on individuals and in other situations with nested data (e.g., visits nested within participants, children nested within schools; siblings nested within families). Note, the ABCD Study has multiple levels of nesting, depending on the analysis, including within-participant, within-family, within-school, within-MRI scanner, and within-site.

Statistical models for nested data include two main components, coupling a model for the mean response and its dependence on covariates with a model for the covariance among repeated outcomes on an individual. In contrast, traditional methods, such as multiple regression and ANOVAs, assume residuals are independent and thus are generally inappropriate for designs that incorporate some type of nesting. Specifically, given that residuals are no longer independent in a repeated measures design, standard errors from these models are biased and can produce misleading inferences. Therefore, an initial question to be addressed by a researcher analyzing prospective data is how to best model their covariance structure. A range of methods can be used to model covariance structures, each with its own set of tradeoffs between model fit and parsimony and which may be more or less appropriate for each specific application (e.g., see Kincaid, 2005).

The most common approach is to use random effects. Essentially, random effects allow for covariance estimates around fixed effects. A classic example (from Bryk and Raudenbush 1992; Singer, 1998) involves math achievement measured among students nested within schools. In a basic, intercept-only model with no covariates (i.e., an unconditional growth model), there would be one fixed effect (the grand mean, or intercept, of math achievement), one school random effect (representing variation in the intercept between schools) and the within-school student residuals (variation left over after accounting for fixed and random effects). In this framework, each student’s score would be the sum of the fixed effect (the grand mean), the school random effect and the student’s within-school residual. Assumptions about the variance and covariance components of this model dictate the form of the variance/covariance structure. For example, if we assume the random effects are independent and identically distributed, the implied structure would be compound symmetry, where it is assumed the covariance of any two students in a single school is captured by a school random intercept and the covariance of any two students in different schools is zero. The assumptions of this relatively simple covariance structure can be relaxed depending on the nesting structure of the data, resulting in different covariance structures with additional parameters (see Singer, 1998).

In longitudinal studies, measurement occasions are nested within individuals. Mixed-effect models can be fitted to longitudinal data that couple a model for growth (development) at the mean level with a model for capturing within-individual covariance of assessments. For example, a linear growth model would involve two fixed effects – one for the intercept (the average score when time is coded zero) and one for the linear slope (the change in scores for each unit increase in time). Random effects could include a random effect for intercept, capturing individual variation in scores at the first measurement occasion, and a random effect for the linear slope, capturing individual variation in linear change across additional measurements. Within-individual residuals account for the remaining variation in assessments after accounting for the fixed and random intercepts and slopes. Assumptions regarding the covariation among the random effects also indicate different covariance structures. For example, it is typical to assume that the random intercept and slope components covary, i.e., an individual’s score at an initial measurement relates to the amount of change exhibited across subsequent measurements. Further, particularly in structural equation model forms of this model, it is sometimes assumed that the variance of the residuals varies across assessments (Curran, 2003).

An alternative to random effects is the autoregressive structure, which allows for correlations between repeated assessments to diminish across time. As the name suggests, the structure assumes the residual of a subsequent measurement occasion (e.g., measurement 2) is regressed onto the residual of a prior measurement occasion (e.g., baseline measurement). The most common type of autoregressive structure is the AR(1), where residuals at time t + 1 are regressed on residuals at time t. Identical to compound symmetry, this model assumes the variances are homogenous across time; however, it differs from compound symmetry in that the correlations between repeated assessments decline exponentially across measurement occasions rather than remaining constant. That is, we can think of the underlying process as a stochastic one that wears itself out over time. For example, per the AR(1) structure, if the correlation between data obtained at the first and second measurement occasions is thought to be.5, then the correlation between first and third measurement occasions would be assumed to be.5 × .5 = .25, while the correlation between first and fourth measurement occasions would be assumed to be.5 × .5 × .5 = .125. As with compound symmetry, the basic AR(1) model is parsimonious in that it only requires two parameters: the variance of the residuals and the autoregressive coefficient.

Notably, the assumption of constant autoregressive relations between assessments is often relaxed in commonly employed designs that use autoregressive modeling (e.g., CLPM). These designs still typically assume an AR(1) process. However, the magnitude of these relations is often allowed to differ across different AR(1) pairs of assessment (e.g., the relation between measurement 1 and measurement 2 can be different from the relation between measurement 2 and measurement 3). These models also often relax the assumption of equal variances of the repeated assessments.

Although the AR(1) structure may involve a more realistic set of assumptions compared to compound symmetry, in that the AR(1) model allows for diminishing correlations across time, the basic AR(1) model, as well as autoregressive models more generally, can also suffer from several limitations in contexts that are common in prospective designs. In particular, recent work demonstrates that if a construct being assessed prospectively across time is trait-like in nature, then a simple AR(1) process fail to adequately account for this trait-like structure, with the downstream consequence that estimates derived from models based on AR structures (such as the CLPM) can be misleading and fail to adequately demarcate between- vs. within-person sources of variance (Hamaker et al., 2015). Note also, discrete-time autoregressive structures such as AR(1) implicitly assumes relatively constant time gaps between measurements; this may not be true in many applications using the ABCD Study data.

#### 3.1.7 Missing data/attrition

Attrition from a longitudinal study such as ABCD is inevitable and represents a potential threat to the external validity of analyses conducted at later visits, especially since attrition can only be expected to grow over time (Littlefield et al., 2022). The ABCD Retention Workgroup employs a data-driven approach to examine, track, and intervene in these issues and while preliminary findings show participant race and parent education level to be associated with late and missing visits, although to date, formal attrition in ABCD has been minimal (Ewing et al., 2022). Ideally, one tries to minimize attrition through good retention practices from the outset via strategies designed to maintain engagement in the project (Cotter et al., 2005, Hill et al., 2016, Watson et al., 2018). However, even the best-executed studies need to anticipate growing attrition over the length of the study and implement analytic strategies designed to provide the most valid inferences.

Perhaps the most key concern when dealing with data that is missing due to attrition is determining the degree of bias in retained variables that is a consequence of attrition. Such bias can attenuate generalizability, particularly if the pattern of missingness is not random (e.g., certain subsets of the population are more likely to drop out/not attend a visit). Assuming that the data are not missing completely at random, attention to the nature of the missingness and employing techniques designed to mitigate attrition-related biases need to be considered in all longitudinal analyses.

Three types of missingness are considered in the literature (Little and Rubin, 1989, Little, 2013), namely: (a) missing completely at random (MCAR), (b) missing at random (MAR), and (c) missing not at random (MNAR). Data that are MCAR are a simple random sample of all data in a given dataset. MAR implies missing data are a random sample (i.e., does not hinge on some unmeasured variables) within strata of the measured covariates in a dataset (e.g., biological sex). Data that are MNAR are missing as a function of unobserved variables and may bias associations even after conditioning on the observed covariates. Graham (2009) provides an excellent and easy-to-digest overview of further details involving missing data considerations.

Modern approaches for handling missing data, such as full-information maximum likelihood, propensity weighting, auxiliary variables and multiple imputation avoid the biases of older approaches (see Enders, 2010; Graham, 2009). Graham (2009) noted several “myths” regarding missing data. For example, Graham notes many assume the data must be minimally MAR to permit estimating procedures (such as maximum likelihood or multiple imputation) compared to other, more traditional approaches (e.g., using only complete case data). Violations of MAR impact both traditional and more modern data estimation procedures, though as noted by Graham, violations of MAR tend to have a greater effect on older methods. Graham thus suggests that imputing missing data is a better approach compared to listwise deletion in most circumstances, regardless of the model of missingness (i.e., MCAR, MAR, MNAR; see Graham, 2009; but also see Twisk et al., 2013). The ABCD Biostatistics Workgroup is currently implementing several missing data approaches which are being implemented and compared to each other (and listwise deletion) in the 6.0 data release, including, propensity score weighting, and multiple (multilevel) imputation.

#### 3.1.8 Impact of COVID-19 on study design and data interpretation

The COVID-19 pandemic has introduced significant challenges to longitudinal studies like the ABCD Study, including disruptions to data collection and alterations in adolescent behavior, mental health, and development. These disruptions could confound longitudinal analyses if not properly addressed, potentially introducing biases such as period effects, which are time-specific events that affect all individuals in a population at a particular point in time. Period effects, such as changes in public health policies or economic downturns, can influence developmental outcomes across cohorts (see Jager et al., 2021). Indeed some recent studies using the ABCD dataset suggest the pandemic's influence on various developmental outcomes (Pelham et al., 2021, Hamatani et al., 2022, Stinson et al., 2021). Moreover, the pandemic's impact on study retention and data collection practices may exacerbate issues related to missing data, particularly increasing the likelihood of data being missing not at random (MNAR). To mitigate these effects, researchers should consider incorporating pandemic-specific covariates and employ analytic techniques such as sensitivity analyses to explore the robustness of findings (Yip et al., 2022, Nagata et al., 2022).

#### 3.1.9 Selection and modeling of covariates

An essential aspect of longitudinal analysis is the careful selection and modeling of covariates, as these choices can significantly influence study results and interpretations. In the context of the ABCD Study, covariates can be categorized as either time-invariant (e.g., demographic variables such as race, ethnicity, and sex) or time-varying (e.g., measures of stress, family environment, or substance use over time). Time-invariant covariates are typically measured at baseline and do not change over the study period, making them useful for controlling for stable characteristics that may influence outcomes. Conversely, time-varying covariates, which are repeatedly measured throughout the study, allow for the examination of how changes in these variables correlate with changes in outcomes over time (Fitzmaurice et al., 2012; Singer & Willett, 2003).

When selecting covariates, it is crucial to base decisions on both the theoretical framework guiding the study and empirical evidence from prior research, alongside appropriate statistical modeling techniques. Theoretical frameworks can help differentiate between confounders, mediators, and other variables that may influence the relationships being studied. This can be critical, as for example, including a mediator as a covariate may obscure causal pathways (van Zwieten et al., 2022). Modeling approaches, such as mixed-effects models are particularly well-suited for longitudinal data, as they can accommodate both fixed effects (time-invariant covariates) and random effects (time-varying covariates), offering a nuanced understanding of how different covariates influence the outcome over time. Moreover, while covariate adjustment aims to control for confounding variables, improper or excessive adjustment can distort relationships, mask true associations, and lead to biased estimates (Meehl, 1971, Lynam et al., 2006).

One recommended approach for covariate selection is to compare a minimally adjusted model with a small set of essential covariates to a fully adjusted model that includes all potential confounders and covariates (van Zwieten et al., 2022). The minimally adjusted model can help assess the baseline relationship between the predictors and outcomes, while the fully adjusted model controls for additional variables. This comparison provides insight into whether over-adjustment might be distorting the results. Researchers should be cautious when adding more covariates to a model, as doing so can sometimes lead to diminishing returns in terms of model precision and reliability. Additionally, performing sensitivity analyses to test the robustness of the results in both the minimally adjusted and fully adjusted models can help identify any significant changes or potential biases introduced by including specific covariates.

Thoughtful selection and modeling of covariates, as emphasized by Saragosa-Harris et al. (2022), is essential for enhancing the robustness and interpretability of results, ultimately leading to more accurate conclusions regarding developmental trajectories. Failure to recognize the appropriate relationships between covariates can lead to misleading conclusions, introducing biases that distort the true dynamics at play. Therefore, covariates must be carefully handled to ensure that observed relationships accurately reflect the underlying processes.

#### 3.1.10 Quantifying effect sizes longitudinally

Given that longitudinal data involve multiple sources of variation, quantifying effect sizes longitudinally is more complex compared to deriving such estimates from cross-sectional data. An effect size can be defined as, “a population parameter (estimated in a sample) encapsulating the practical or clinical importance of a phenomenon under study.” (Kraemer, 2014). Common effect size metrics include the Pearson correlation r between two variables and the standardized difference between two means, Cohen’s d (Cohen, 1988). An extensive discussion of cross-sectional effect sizes and their relevance for ABCD is given in Dick et al. (2021).

Adjustments to common effect size calculations, such as Cohen’s d, are required even when only two time points are considered (e.g., Morris and DeShon (2002). Wang et al. (2019) note there are multiple approaches to obtaining standardized within-person effects, and that commonly suggested approaches (e.g., global standardization) can be problematic (see Wang et al., 2019, for more details). Thus, obtaining effect size metrics based on standardized estimates that are relatively simple in cross-sectional data (such as r) becomes more complex in the context of prospective longitudinal data. Feingold (2009) noted that equations for effects sizes used in studies involving growth modeling analysis (e.g., latent growth curve modeling) were not mathematically equivalent, and the effect sizes were not in the same metric as effect sizes from cross-sectional analysis (see Feingold, 2009, for more details).

Given this issue, there have been various proposals for adjusting effect size measures in repeated assessments. Feingold (2019) reviews the approach for effect size metrics for analyses based on growth modeling, including when considering linear and non-linear (e.g., quadratic) growth factors. Morris and DeShon (2002) review various equations for effect size calculations relevant to combining estimates in meta-analysis with repeated measures and independent-groups designs. Other approaches to quantifying effect sizes longitudinally may be based on standardized estimates from models that more optimally disentangle between- and within-person sources of variance. As an example, within a random-intercept cross-lagged panel model (RI-CLPM) framework, standardized estimates between random intercepts (i.e., the correlation between two random intercepts for two different constructs assessed repeatedly) could be used to index the between-person relation, whereas standardized estimates among the structured residuals could be used as informing the effect sizes of within-person relationships.

#### 3.1.11 Longitudinal data structures

An ideal longitudinal analysis integrates (a) a well-articulated theoretical model, (b) an appropriate longitudinal data structure, and (c) a statistical model that is an operationalization of the theoretical model (Collins, 2006). To accommodate various research questions and contexts, different types of longitudinal data and data structures have emerged (see Fig. 1). An understanding of these data structures is helpful, as they can warrant different types of LDA. Given that identifying a starting point for making comparisons is somewhat arbitrary, Curran and Bauer (2019) provide a nice on-ramp in first distinguishing between the use of “time-to-event” and “repeated measures” data. Although both model time, the former is concerned with whether and when an event occurs, whereas the later is focused on growth and change (Curran and Bauer 2019). Time-to-event structures measure time from a well-defined origin point up to the occurrence of an event of interest. This data structure is most often analyzed using survival analysis methods (e.g., hazard rate models, event history analysis, failure-time models and the time-to-event data can be based on a single assessment or include multiple recurrent or competing events). While much has been written about “time-to-event” data (Hosmer et al., 2008, Rizopoulos, 2012), including a recent analysis examining exclusionary discipline in schools using data from the ABCD Study (Brislin et al., 2024), our emphasis will be given to the modeling of “repeated measures” data.

**Figure:**
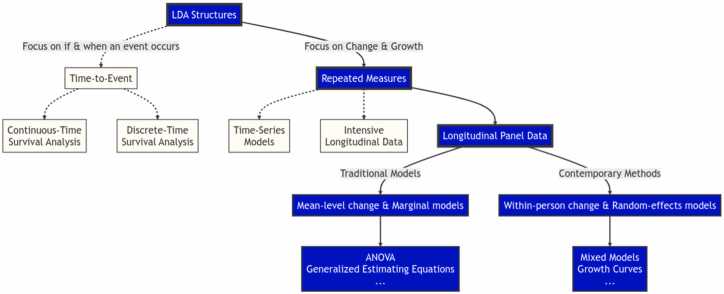

When discussing longitudinal analysis, we are most often talking about data collected on the same unit (e.g., individuals) across multiple measurement occasions. However, repeated-measures analysis is not a monolith, and it will serve us well to distinguish between a few of the most common types. One such approach to repeated measures analysis is the use of time-series models. These models generally consist of a long sequence of repeated measurements (≧ 50–100 measurements) on a single or small number of variables of interest. Time-series analysis is often used to predict temporal trends and cyclic patterns and is geared toward making inferences about prospective outcomes within a population (with relatively less focus on inferring individual-level mechanisms and risk factors).

A related type of repeated measures analysis is Intensive Longitudinal Data (ILD) (Curran and Bauer, 2011). Similar to time-series analysis, ILD models involve frequent measurements (approximately 30–40 measurements) of the same individuals within a relatively circumscribed period (e.g., experience sampling to obtain time series data from many individuals). Although ILD models may include slightly fewer measurement occasions than traditional time-series data, they typically involve a larger number of subjects (around 50–100 subjects), which enables the examination of short-term patterns across a more representative sample (Bolger and Laurenceau, 2013). ILD models are particularly valuable for capturing dynamic processes and can incorporate time-series modeling techniques that fit parameter estimates to each individual’s data, thus allowing for the exploration of individual differences in outcomes (Curran and Bauer, 2011). With the growing use of ILD in fields like neuroimaging and behavioral science, advanced analytical techniques such as Dynamic Structural Equation Modeling (DSEM) (Asparouhov et al., 2018), Group Iterative Multiple Model Estimation (GIMME) (Gates and Molenaar, 2012), and Unified Structural Equation Modeling (uSEM) (Gates et al., 2010) are becoming increasingly relevant. DSEM, for instance, extends traditional SEM by allowing for the modeling of time-varying processes and latent variables within ILD, making it possible to disentangle within-person and between-person variations over time (Asparouhov et al., 2018). GIMME offers a data-driven approach to identify group-level patterns while accounting for individual-level heterogeneity, which is particularly useful when working with datasets that involve trial-level or mobile data (Gates and Molenaar, 2012). uSEM, on the other hand, integrates multiple types of data (e.g., time-series, multilevel, and latent variable models) within a unified framework, offering a flexible approach to model complex, dynamic processes in ILD (Gates et al., 2010). The application of these techniques to ABCD data not only enhances the ability to model developmental trajectories with greater precision but also opens avenues for integrating multiple data sources, such as neuroimaging, behavioral, and mobile data. These methods provide powerful tools for researchers to investigate the interplay between various factors affecting adolescent development, offering deeper insights into the mechanisms underlying observed behaviors and outcomes.

The final type of repeated measures analysis that we will primarily focus on is the longitudinal panel study. These models follow a group of individuals— a panel (also referred to as a cohort) — across relatively fewer measurement occasions (∼ 5–15) and are often focused on examining both change within- and between-individuals. The ABCD Study is primarily a longitudinal panel study, though some data streams (e.g., functional brain imaging, FitBit data) could be analyzed as ILP or even time series methods.

While other longitudinal designs have their own unique strengths and applications, the longitudinal panel design is particularly well-suited for investigating developmental processes in the context of the ABCD Study. In the following sections, we will discuss various analytic methods commonly used to analyze longitudinal panel data, including growth models, mixed models, and a number of additional trajectory models. These methods provide valuable insights into within- and between-individual differences and are highly relevant for researchers working with the ABCD Study dataset. By focusing on these methods, we aim to equip readers with the knowledge necessary to conduct longitudinal research and perform analyses using the rich, longitudinal, and publicly available data from the ABCD Study.

## 4 Longitudinal analysis

### 4.1 Types of longitudinal panel models

With the large and continually expanding body of research on statistical methods for longitudinal analyses, determining which longitudinal model to implement can be challenging. This section aims to help researchers navigate these many options to identify the statistical approach most appropriate to their unique research question when deciding on how to measure change over time. Notably, there are a myriad of viable ways one can go about grouping various types of longitudinal models for presentation.

**Fig. 2:**
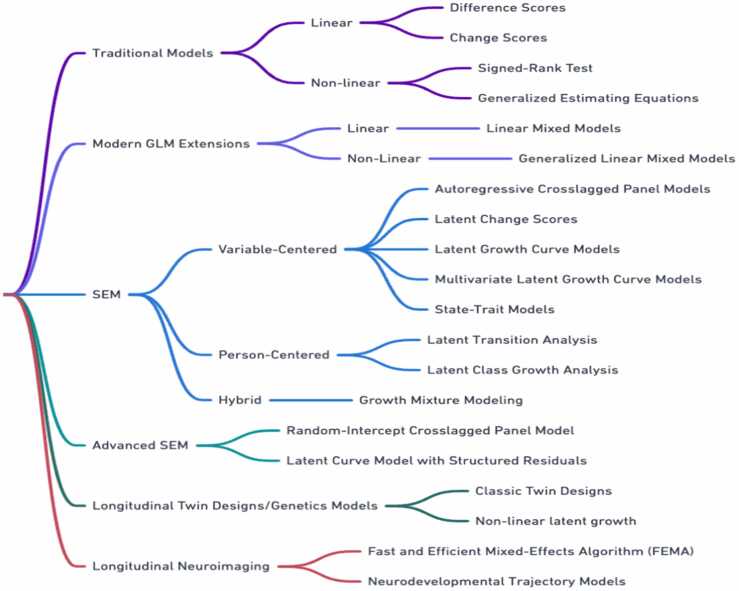
Longitudinal Models/Data Structures.

Common examples include grouping by linear vs nonlinear models (Collins, 2006), the number of measurement occasions (King et al., 2018), and statistical equivalency (e.g., change scores vs. residualized change; see Castro-Schilo and Grimm, 2018). The organization we use below overlaps in several ways with these examples, and in particular with Bauer and Curran (2019). However, it is important to note that in each case, the chosen way of grouping is primarily intended to allow the reader to compare and contrast various analytical approaches (see McCormick et al., 2023). In the following sections, we briefly summarize the advantages/disadvantages of a series of longitudinal models organized into the following groupings: Traditional Models, Modern GLM Extensions, Structural Equation Models (SEM), and Advanced SEM (see Fig. 2). We note that this is not an exhaustive review of each of these methods, and for more in-depth detail we do provide the reader with relevant resources. As aptly summarized by Bauer and Curran (2019), “…there are many exceptions, alternatives, nuances, ‘what ifs’, and ’but couldn’t you’s that aren’t addressed here.”

### 4.2 Traditional models

Traditional methods for longitudinal analysis primarily focus on modeling mean-level change, and how these changes may differ across groups or levels of some other variable. For example, is there a difference in average internalizing symptoms obtained across multiple assessments between boys and girls? Longitudinal models that focus on mean-level change are also referred to as marginal models and examples of specific methods include repeated measures ANOVA, ANCOVA and Generalized Estimating Equations (GEEs). Mean-level change models are commonly used when data are only available from 2 measurement occasions. For example, computing a difference score (e.g., mean internalizing scores at visit 2 - mean internalizing scores at visit 1) that can be used as an outcome in a subsequent GLM analysis (e.g., paired-samples *t*-test, repeated measures ANOVA) to test for differences in patterns of change over time and between groups. Additionally, the longitudinal signed-rank test, a nonparametric alternative to the paired *t*-test, can be a useful tool for analyzing non-normal paired data. Another common approach, often used in pre-/post-design studies but can be used with ABCD Study data, is to use residualized change score analysis to assess the degree of change in a variable, while controlling for its initial level (Castro-Schilo and Grimm, 2018).

For example, to examine change in cortico-limbic connectivity among ABCD participants, (Brieant et al., 2021) regressed cortico-limbic connectivity at the year 2 follow-up on baseline cortico-limbic connectivity, which allowed the authors to examine the associations between negative life events and the variance of cortico-limbic connectivity unexplained by baseline connectivity. Similarly, Romer and Pizzagalli (2021) used a residualized-change model to examine the bidirectional influences of executive functioning and a general psychopathology factor ‘p’ across the first two years of the ABCD Study. Both studies were able to conclude associations between their constructs of interest that could not be accounted for by prior frequencies at baseline.

Traditional longitudinal models, such as residualized change score models, can be useful in some contexts (e.g., two measurement occasions), but overall, their practical utility for answering questions about developmental processes is limited. Perhaps most notably, these models do not allow for characterizing patterns of within-person change. This is a particularly important limitation since most psychological theories posit within-person processes (i.e., what will happen within a given individual). As such, traditional approaches often correspond poorly with most theoretical models of change and a failure to disaggregate between-person and within-person effects can result in consequential errors of inference (e.g., ecological fallacy; Curran and Bauer, 2011). Moreover, even determining which of these procedures to use for comparing change over two time points across groups can be surprisingly complicated. A particularly vexing example is that of imbalanced baseline scores (i.e., when baseline scores are correlated with a covariate of interest), which can produce different conclusions across methods (e.g., see Littlefield, 2023, for a review). Given these shortcomings, and the complexity of the issues surrounding some of these methods, it is typically recommended that researchers make use of more modern approaches for analyzing longitudinal data and preferably make use of data collected across three or more time points, as is currently true for many ABCD Study assessments.

### 4.3 Modern GLM extensions

Modern approaches to LDA have advanced beyond traditional methods by offering greater flexibility and a more in-depth understanding of within-person and between-person variability. Generalized Estimating Equations (GEE), Linear Mixed Models (LMM), Generalized Linear Mixed Models (GLMM), and Autoregressive Cross-Lagged Panel Models (ARCL) are examples of such contemporary techniques. GEE, an extension of Generalized Linear Models, combines the generalized linear model for non-normal outcomes with repeated measures and is suitable for analyzing correlated longitudinal data and modeling population-averaged effects. For example, Van Dijk et al. (2021) used GEE to obtain relative risks for psychiatric diagnoses among children in the ABCD Study with a family history of depression and used the ABCD Study sampling weights to generalize prevalence rates among 9 and 10-year-olds across the US.

LMMs, also known as multilevel or hierarchical linear models, facilitate the simultaneous analysis of within-person and between-person variability, making them ideal for nested data structures or repeated measures. Within the ABCD Study, researchers may want to consider nesting by individual, family (i.e., siblings or twins), school or district, and/or site. GLMMs further extend the LMM framework to accommodate non-normal response variables, such as binary, count, or ordinal data, such as the use of ABCD data on substance use (e.g., Martz et al., 2022), screen media use (Lees et al., 2020), and microstructure of the brain (Palmer et al., 2022).

Finally, ARCL models are used to investigate reciprocal relationships between variables over time, as they estimate both autoregressive and cross-lagged effects, although ARCL models are relatively less useful for teasing apart between-person and within-person sources of variances; see (Curran and Hancock, 2021).

The strengths of these modern methods lie in their ability to account for individual differences, within-person change, and time-varying predictors, thereby providing a more comprehensive understanding of complex relationships in longitudinal data. Despite these advantages, modern approaches may require more complex modeling assumptions and higher computational demands compared to traditional methods. Additionally, proper model specification and the interpretation of results can be more challenging, especially in cases of high multicollinearity or missing data. However, modern longitudinal analysis methods have generally surpassed traditional methods in addressing a wider range of research questions, accommodating diverse data structures, and elucidating the intricate dynamics of developmental processes.

### 4.4 Structural equation modeling (SEM)

Structural Equation Modeling (SEM) is a flexible modeling framework that integrates elements of path analysis and confirmatory factor analysis (CFA) to examine complex relationships between a set of observable variables and latent constructs (Hair et al., 2021). The integration of structural (regression) and measurement (CFA) components within a unified framework supports a theory-driven approach that allows researchers to rigorously test hypothesized relationships among variables of interest and their underlying causes (Hair Jr et al., 2021, Raykov and Marcoulides, 2012). Over the years, the flexibility of the SEM framework has evolved to become particularly adept for modeling autoregressive processes (which often assume underlying stationarity) and growth processes which accommodate both, mean trajectories and individual differences in them (McArdle, 2009, Little, 2013).

Longitudinal SEM techniques share many similarities with mixed-effects methods and research demonstrates their mathematical equivalence in many situations (Curran, 2003, Mehta and Neale, 2005). However, these related approaches often cater to distinct theoretical and analytical needs. For instance, mixed-effects techniques are an extension of the regression framework and often excel when working with complex data structures such as multiple levels of nesting, small samples, and non-equidistant time points (McNeish and Matta, 2018; Hedeker and Gibbons 2006). Alternatively, applying SEM methods to longitudinal analysis provides a flexible means for modeling the underlying process of change. It also addresses several challenges faced by competing approaches, including the ability to accommodate intricate error structures and deal effectively with missing data, as well as the implementation of numerous modeling extensions (McNeish and Matta, 2018); Curran and Hancock, (2021)). These models have grown increasingly popular for modeling longitudinal outcomes particularly due to their ability to build statistical models that match some particular underlying theory (Serang et al., 2019).

Considering the variety of available techniques, it can be helpful to classify longitudinal SEM, broadly (if not coarsely), into variable-centered, person-centered, and hybrid analyses, each with unique strengths and limitations. Variable-centered analyses (e.g., latent growth curves (Curran, 2003), latent change scores (McArdle and Hamagami, 2001), latent state-trait models (Geiser and Lockhart, 2012) are primarily concerned with understanding covariation among variables at the group level and characterizing population-level patterns of change, while person-centered analyses (e.g., latent class and latent transition models) identify distinct subgroups or patterns within the data (Muthén and Muthén, 2000, Woo et al., 2024, Howard and Hoffman, 2018). Hybrid models combine these perspectives to offer a comprehensive analysis of latent subgroups and growth parameter relationships (Morin et al., 2018, Lubke and Muthén, 2005). The choice between these approaches is primarily driven by the research question, data structure, and relevant underlying assumptions.

### 4.5 Variable-centered models

One key application of the SEM framework to the analysis of longitudinal data is the latent growth curve model (LGCM). This is a variable-centered approach that characterizes average group trajectories and individual variations (random effects) in an outcome over time (Curran, 2003). These models are similar to their linear mixed effects counterpart in many ways, with the main conceptual difference being that LGCM includes a repeatedly measured outcome in the model as a function of time (closely resembling a standard CFA approach), rather than as an explanatory variable (as in a standard regression approach; McNeish and Matta, 2018). Specifically, observed scores at each time point are treated as indicator variables with their factors loading scaled to reflect a hypothesized pattern of change (e.g., loadings of 0, 1, and 2 would assume equidistant, linear change). Latent intercepts (initial levels) and slopes (rates of change) are estimated, along with their variances and covariance to capture common trends and individual deviations over time. This method was used in a recent study by Trevino et al. (2023) to show a decreasing trajectory of parent-reported externalizing behaviors from ages 9–12 among youth taking part in the ABCD Study. This study also examined hypothesized predictors of the growth trajectory intercept and slope factors, highlighting a particular strength of these models–– their flexibility and extensibility. As an example, Roy et al. (2024) used publicly available data from the ABCD Study and several other large-scale datasets to explore bivariate (parallel process) relationships between white matter pathways and literacy over time. Beyond these examples, LGCMs can be extended in numerous ways, including to compare rates of growth across groups, investigate the consequences of change, and incorporate time-invariant or time-varying covariates, to highlight only a few (for a more detailed treatment of LGCM applications and methodologies (refer to Preacher et al., 2010; Preacher, 2018; Curran et al., 2010).

The latent change score model (LCSM) is a variable-centered approach uniquely tailored for analyzing temporal variations in how a construct changes over time (McArdle and Nesselroade, 1994, McArdle and Hamagami, 2001). These models share many features with growth curve analysis, but with a more explicit focus on how change occurs between measurement occasions (Serang et al., 2019, McArdle, 2009). Specifically, LCSM estimates a series of latent variables to model change in an outcome from one time point to the next, as a function of scores on that outcome at prior time points (McArdle and Hamagami, 2001, Ghisletta and McArdle, 2012). Some types of LCSM estimate two underlying latent factors: a constant change factor that remains fixed over time, and a proportional change factor that adjusts for previous scores. By disaggregating change into constant and proportional components, this approach facilitates a more nuanced understanding of whether prior changes in a given process are related to future changes in the same process (Serang et al., 2019, Kievit et al., 2018). Expanding upon the capabilities of this framework, LCSM also allows for comprehensive multivariate analyses that can facilitate investigations into how change in one construct is associated with change in another construct. The appeal of this approach is evidenced by several recent studies that have used data from the ABCD Study to explore bivariate associations between brain development and changes in several mental and physical health indicators (e.g., Wiker et al., 2023; Rapuano et al., 2022; Beck et al., 2023; Nweze et al., 2023; Mewton et al., 2023).

Latent State-Trait Models (LSTM) offer another variable-centered approach to longitudinal analysis that also allows for the estimation of patterns of change over time. Unlike LGCM, which conceptualizes change as a function of time, and LCSM, which views change through sequential measurements, this approach disaggregates observed behaviors into distinct stable (trait) and occasion-specific (state) components (Kenny and Zautra, 2001, Steyer et al., 2015). Based on LST theory (Steyer et al., 1999, Steyer et al., 1992), these models hold that scores on a repeated measures outcome can be partitioned into an enduring latent trait variable that reflects between-individual differences, and a transient latent state residual that represents situational influences (Stadtbaeumer et al., 2022, Geiser and Lockhart, 2012). Beyond parsing out these key variance components, LSTM can be extended in many ways, such as by incorporating autoregressive effects to capture relative stability and the influence of past states on future responses (i.e. carry-over effects; Cole et al., 2005; Eid et al., 2017; Geiser and Lockhart, 2012). The merits of this approach are highlighted in a recent review by Sanchez-Alonso and Aslin (2020) focused on strategies for modeling neurobehavioral development. These study authors encourage researchers to leverage data from the ABCD Study and other large-scale longitudinal and publicly available datasets and to apply state-trait methods to map neural and behavioral trajectories in youth (for a more detailed overview of these models, (see Kenny and Zautra, 2001; Steyer et al., 2023; Steyer et al., 1999). In general, while many commonalities and important features are shared across different variable-centered approaches, selecting the most appropriate statistical model for assessing change hinges on the specific theoretical model of change and what is intended to be learned from the model (see Kievit et al., 2018; McArdle, 2009; Ghisletta and McArdle, 2012, for discussion), which is critical for informing the interpretation and applicability of the research findings.

### 4.6 Person-centered models

Despite the flexibility afforded by variable-centered analysis, these methods are not generally equipped to capture underlying developmental trajectories that are unique to distinct clusters of individuals. This limitation can be particularly notable for research that aims to characterize heterogeneous developmental processes. Person-centered approaches, including latent transition analysis and latent class growth analysis, address this limitation by identifying subgroups of individuals who share similar patterns of change. These models can reveal meaningful subpopulations and help researchers understand the factors that contribute to differences in developmental trajectories. For example, taking advantage of the large sample size of the ABCD Study, Xiang et al. (2022) found evidence of four subgroups of youth with unique longitudinal patterns of depressive symptoms over time and identified risk factors that were differentially associated with the various trajectories.

The use of such models allows for a more nuanced understanding of the associations between risk factors and change in symptomatology, as opposed to a snapshot of symptomatology at a single time point. Despite a range of potential model specifications for longitudinal mixture modeling, person-centered approaches tend to use parameterizations that default to settings found in popular software packages (e.g., Mplus). It has recently been demonstrated (see McNeish and Harring, 2021) that the use of such specifications tends to identify the so-called “cat’s cradle” solution (see Sher, Jackson, and Steinley, 2011) that consists of “…(a) a consistently ‘low’ group, (b) an ‘increase’ group, (c) a ‘decrease’ group, and (d) a consistently ‘high’ group” (Sher et al., 2011, p. 322). Indeed, Xiang et al. (2022) describe their four-group solution as follows: “Of all participants, 536 (10.80 %) were classified as increasing, 269 (5.42 %) as persistently high, 433 (8.73 %) as decreasing, and 3724 (75.05 %) as persistently low” (Xiang et al., 2022, p. 162). Although Sher et al. (2011) cautioned that groups from these trajectory-based approaches should not be over-reified, this practice also remains common (e.g., Hawes et al., 2016; Hawes et al., 2018). Thus, though person-centered approaches can, in theory, help researchers understand the factors that contribute to differences in developmental trajectories, researchers should more thoughtfully consider alternative specifications (see Littlefield et al., 2010, as an example) and be especially skeptical when default specifications identify these four prototypic groups.

Hybrid approaches, such as growth mixture (jung 2008; Muthén and Muthén, 2000) and factor-mixture (Lubke and Muthén, 2005, Lubke and Muthén, 2007) modeling, combine aspects of both variable-centered and person-centered models, allowing for the identification of latent subgroups while also modeling relationships among growth parameters. This combination provides a more comprehensive understanding of longitudinal data by capturing both within- and between-person variability. However, hybrid models can be more complex, necessitating careful model specification, selection, and interpretation. Additionally, these methods may require larger sample sizes to ensure the stability and accuracy of results.

In summary, SEM approaches offer powerful tools for LDA, enabling researchers to investigate complex relationships, individual differences, and change dynamics over time. The choice between variable-centered, person-centered, and hybrid approaches depends on the research objectives and the nature of the data. Despite their limitations, these models have greatly advanced our understanding of developmental processes and the factors that contribute to individual differences in change trajectories.

### 4.7 Advanced structural equation models

Advanced SEM approaches, such as the RI-CLPM and LCM-SR models, have emerged to provide a clearer understanding of important research questions and data structures in longitudinal analysis. These advanced models extend traditional SEM techniques, enabling researchers to disentangle within-person and between-person effects, as well as capture additional time-specific dependencies and associations that may not be accounted for by the latent growth factors.

The RI-CLPM enhances the traditional cross-lagged panel model by incorporating random intercepts, which allow for the separation of stable individual differences from the dynamic within-person associations between variables over time. Within-person variance in these models is captured by a series of latent variables that reflect time specific variance (i.e., the residual variance from the random intercept). These time-specific variables are referred to as structured residuals. Distinguishing between-person variance subsumed by the random intercept from the structured residuals is particularly valuable for understanding the time-specific effects of one variable on another, while accounting for the influence of individual differences. However, RI-CLPM may require larger sample sizes to ensure stability and accuracy of the estimates and can be computationally demanding. Using three waves of ABCD Study data, Kulisch et al. (2023) found a prospective association between psychopathology and childhood obesity as well as between childhood obesity and later eating behavior. The authors also showed that reciprocal associations were overestimated when stable, interindividual trait differences were not included in the model (i.e., via the random intercept).

LCM-SR, on the other hand, extends the RI-CLPM by including additional growth factors, such as a random linear slope. That is, the LCM-SR is a hybrid between a latent growth model and CLPM. This approach allows for a more comprehensive understanding of within-person change dynamics and factors influencing change over time. By including structured residuals, LCM-SR can capture additional time-specific relationships that are not explained by the latent growth factors. However, even more so than the RI-CLPM, LCM-SR comes with increased model complexity and requires careful specification and interpretation.

In conclusion, advanced SEM approaches for LDA provide valuable tools for addressing complex research questions and data structures. While they offer more nuanced insights into within-person change dynamics and the influence of individual differences, these models also come with certain limitations, such as the necessity of multiple assessments (e.g., four or more for LCM-SR), increased complexity, computational demands, and the need for careful model specification and interpretation. As with any statistical method, researchers should carefully consider their research objectives, data characteristics, and the assumptions of each model when selecting the most appropriate advanced SEM approach for longitudinal analysis. Given that these modeling approaches necessitate more waves of data, they are not yet commonly used with ABCD Study data. We anticipate that as more waves of ABCD data are publically released, these models can be used to address some of the pitfalls of the more traditional methods.

### 4.8 Longitudinal analysis of neuroimaging data

**Fig. 3:**
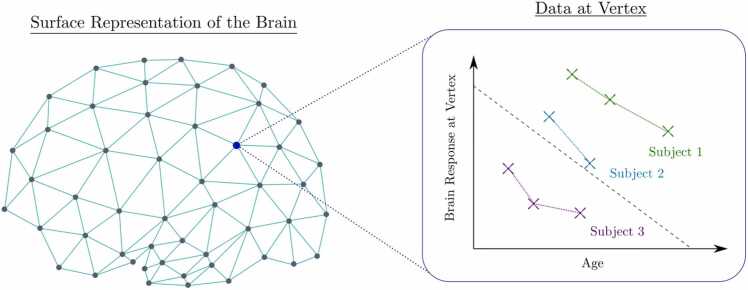
Conceptual Illustration of Longitudinal Neuroimaging Analysis and Statistical Modeling Approaches.

Neuroimaging data, characterized by its large scale, spatial structure and binary data formats, requires the use of specialized software for effective analysis. Fortunately, there are now several freely available software packages that provide options for statistical modeling of brain imaging data, thus facilitating analysis of the brain’s function or structure at every voxel or vertex in an image (see Fig. 3). However, the most widely used packages have only rudimentary support for longitudinal data. Prominent software solutions such as SPM (https://www.fil.ion.ucl.ac.uk/spm) and FSL (https://fsl.fmrib.ox.ac.uk) packages offer limited support in analyzing longitudinal data due to their use of strong assumptions. SPM assumes a common longitudinal correlation structure over space, and FSL requires balanced designs and relies on the assumption of compound symmetry. Despite these limitations, there is a steady growth in the development of neuroimaging tools that provide for comprehensive longitudinal data analysis, typically via commonly adopted modeling approaches such as LMMs marginal models. These emerging tools are equipped to handle more complex scenarios, including unbalanced designs and random covariate effects, among others.

AFNI (https://afni.nimh.nih.gov/), a well-established tool in neuroimaging, integrates 3dLMEr Chen et al. (2013); https://afni.nimh.nih.gov/pub/dist/doc/program_help/3dLMEr.html which adopts an LMM-based approach by providing access to the advanced capabilities of R’s lme4’s lmer function. For surface-based data, Freesurfer (https://surfer.nmr.mgh.harvard.edu/) provides the linear mixed effects (LME) package for modeling longitudinal data (https://surfer.nmr.mgh.harvard.edu/fswiki/LinearMixedEffectsModels). This tool is able to apply spatial regularization of LMM parameters with surface-based ROIs to improve stability (Bernal-Rusiel, Reuter et al., 2013).

For imaging data, LMM’s present a significant computational challenge, not only because they require iterative optimization but also because the computations cannot be vectorized as efficiently compared to ordinary least squares. To overcome this challenge, The Big Linear Mixed Models (BLMM, https://github.com/NISOx-BDI/BLMM) software addresses this by using Python’s broadcasting operations to estimate LMM’s as efficiently as possible (Maullin-Sapey and Nichols, 2022, Maullin-Sapey and Nichols, 2021). BLMM further separates the computation of sufficient statistics and parameter estimation, allowing sensitive image data to remain private if needed.

A different yet efficient approach is used with Fast and Efficient Mixed-effects Analysis (FEMA, https://github.com/cmig-research-group/cmig_tools), which uses a non-iterative regression estimator of the LMM variance components plus variance parameter quantization. This allows vectorization within groups of voxels that share the same variance parameters (Parekh et al., 2021). While this method uses different approximations, the authors have shown it provides results that closely match a traditional LMM implementation.

An alternative method for modeling longitudinal data is the marginal model. This approach differs from others by modeling only the population-level factors and covariates rather than explicitly modeling individual intercepts and slopes. It employs a flexible intra-subject covariance model to account for residual dependence. Like the LMM, it allows for unbalanced designs and singleton subjects, and it implicitly measures any covariance that would otherwise be explained by random covariates. In a marginal model, a “working covariance” matrix is utilized. This matrix does not need to be accurately modeled and may even be constructed under the potentially incorrect assumption that the errors are independent. In this case, parameter estimation reduces to ordinary least squares, but remains consistent. A crucial component of this method is the use of a robust “sandwich estimator” for standard errors, which accounts for any dependence not captured by the working covariance. The Sandwich Estimator (SwE) is a toolbox for SPM (https://www.nisox.org/Software/SwE, including CIFTI support) and FSL (https://fsl.fmrib.ox.ac.uk/fsl/fslwiki/Swe) that provides marginal model inference using an independence working covariance matrix (Guillaume et al., 2014).

**Figure:**
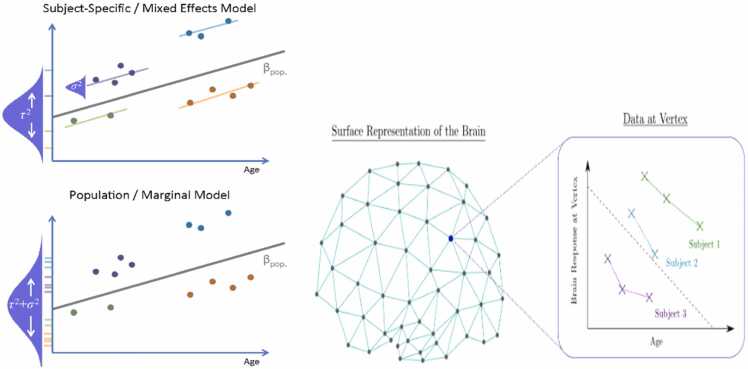

## 5 Discussion

As we enter the era of large-scale longitudinal investigations, it is essential to critically examine the various analytical methods that can be employed to glean insights from these rich datasets. The complex nature of longitudinal data demands sophisticated and well-suited methodologies to accurately address research questions and minimize biases. This paper aimed to provide an overview of diverse longitudinal analysis techniques, with a particular emphasis on their application to extensive longitudinal studies such as the ABCD Study. Beyond contributing to the ever-growing body of knowledge on LDA, we hope this manuscript also serves as a valuable resource for researchers seeking to optimize the use of large-scale longitudinal investigations in advancing our understanding of human development and behavior. In this discussion, we will focus on the key findings and recommendations of our review and discuss potential innovations that can further enhance the utility of these methods.

We began by addressing fundamental concepts and considerations in longitudinal research that are essential for generating accurate and meaningful insights into developmental processes. Concepts such as vulnerable periods, developmental disturbances and snares, or cascade and experience effects (among many others), are instrumental in shaping the design, analysis, and interpretation of longitudinal studies. Together, these concepts provide a framework for understanding the mechanisms underlying the course of development, while also accounting for the complex interplay between individual development and the influence of environmental factors. By considering the intricate relationships among these factors, researchers can better identify the critical time periods, situations, and contexts that contribute to individual differences in developmental outcomes. This awareness enables more precise inferences regarding the causal relationships between exposures and outcomes, ultimately leading to more robust and meaningful findings that can help facilitate the translation of research findings into practical applications in clinical and public health settings.

We also discussed some of the opportunities, challenges, and pitfalls that arise when working with longitudinal data. Key issues include selecting appropriate methods to account for the intricacies of longitudinal data, addressing missing data in a way that minimizes biases, and determining suitable longitudinal data structures that align with research questions and context. To address these challenges, researchers should carefully consider issues such as study design, selection of methods that account for both within- and between-person sources of variance, and employing modern techniques, (e.g., FIML, multiple imputation) for handling missing data. By adhering to best practices in longitudinal research and remaining vigilant of potential pitfalls, researchers can effectively harness the power of longitudinal data to maximize the potential of their investigations and gain valuable insights into complex developmental processes, individual differences, and the underlying mechanisms that drive change over time.

The adoption of open science practices, including the sharing of analysis code and worked examples, is increasingly recognized as essential for advancing the transparency and reproducibility of research, especially in complex areas such as longitudinal modeling. By making code available, researchers enable others to scrutinize, replicate, and build upon their work, thereby fostering a clearer understanding of how different types of longitudinal models are constructed and applied. This practice is particularly beneficial in large-scale studies like the ABCD Study, where multiple research teams may analyze the same dataset using varying methodologies. Code sharing allows for direct comparison of approaches, helping to elucidate why different analyses of the same data might yield divergent results.

In addition to code sharing, it is crucial for researchers to provide comprehensive details about their modeling choices. This includes specifying the type of longitudinal model used (e.g., mixed-effects models, growth curve models, or DSEM), the selection of covariates, and the handling of missing data, as well as any assumptions or constraints applied during the analysis. Clear reporting of these aspects ensures that other researchers can accurately interpret the findings and replicate the study if necessary. By embracing these open science practices, the research community can work towards more robust, transparent, and replicable longitudinal research, ultimately leading to stronger and more reliable scientific conclusions.

The final section, along with associated code and additional resources made available online, aims to serve as a resource for researchers seeking to understand and implement various longitudinal panel models. By providing an overview of different approaches, their strengths and limitations, and key considerations for their use, we hope to facilitate the selection of appropriate models tailored to specific research questions and data structures. It is essential for researchers to consider their research objectives, the characteristics of their data, and the assumptions underlying each model when choosing the most suitable approach for longitudinal analysis.

We encourage researchers to consult the cited literature and online materials for further guidance in selecting and implementing longitudinal models when using the ABCD Study dataset. As the field continues to advance, we anticipate the emergence of new methods and refinements to existing approaches, further expanding the toolkit available to researchers for the analysis of longitudinal data. By staying informed about developments in this area and critically evaluating the appropriateness of different models for their research questions, researchers can ensure that their longitudinal analyses are both rigorous and informative. Notably, in this vast and continually evolving field, with numerous models and approaches available to address a wide range of research questions, no single model is universally applicable or without limitations. The diversity of methods ensures that researchers can find an appropriate tool for their specific needs. By familiarizing themselves with the various types of longitudinal models, researchers can more effectively navigate the complexities of longitudinal data and contribute valuable insights into the developmental processes and individual differences that shape human experience.

## CRediT authorship contribution statement

**Bayat Mohammadreza:** Writing – review & editing, Writing – original draft, Methodology. **Byrd Amy L.:** Writing – review & editing, Writing – original draft, Methodology, Conceptualization. **Castro-de-Araujo Luis FS:** Writing – review & editing, Writing – original draft, Conceptualization. **Dick Anthony:** Writing – review & editing, Writing – original draft, Methodology, Investigation, Conceptualization. **Heeringa Steven F.:** Writing – review & editing, Writing – original draft, Methodology, Conceptualization. **Kaiver Christine M.:** Writing – review & editing, Writing – original draft. **Lehman Sarah M.:** Writing – review & editing, Writing – original draft. **Li Lin:** Writing – review & editing, Writing – original draft, Methodology. **Linkersdörfer Janosch:** Supervision, Methodology, Investigation, Conceptualization. **Maullin-Sapey Thomas J.:** Writing – review & editing, Writing – original draft, Conceptualization. **Neale Michael C.:** Writing – review & editing, Writing – original draft, Methodology, Conceptualization. **Nichols Thomas E.:** Writing – review & editing, Writing – original draft, Methodology, Conceptualization. **Perlstein Samantha:** Writing – review & editing, Writing – original draft. **Tapert Susan F.:** Writing – review & editing, Writing – original draft. **Vize Colin E.:** Writing – review & editing, Writing – original draft. **Hawes Samuel W.:** Writing – review & editing, Writing – original draft, Supervision, Software, Project administration, Methodology, Conceptualization. **Wagner Margot:** Writing – review & editing, Writing – original draft, Project administration, Methodology. **Littlefield Andrew K.:** Writing – review & editing, Writing – original draft, Supervision, Project administration, Methodology, Investigation, Conceptualization. **Waller Rebecca:** Writing – review & editing, Writing – original draft, Methodology, Conceptualization. **Lopez Daniel A.:** Writing – review & editing, Writing – original draft, Visualization, Project administration, Methodology, Investigation, Conceptualization. **Thompson Wesley K.:** Writing – review & editing, Writing – original draft, Supervision, Project administration, Methodology, Investigation, Conceptualization. **Sher Kenneth J.:** Writing – review & editing, Writing – original draft, Supervision, Project administration, Methodology, Investigation, Conceptualization. **Thompson Erin L.:** Writing – review & editing, Writing – original draft, Project administration, Methodology, Conceptualization. **Gonzalez Raul:** Writing – review & editing, Writing – original draft, Supervision, Investigation, Conceptualization. **Aguinaldo Laika D.:** Writing – review & editing, Writing – original draft, Conceptualization. **Adams Ashley R.:** Writing – review & editing, Writing – original draft.

## Declaration of Competing Interest

The authors declare that they have no known competing financial interests or personal relationships that could have appeared to influence the work reported in this paper.

## Data Availability

All code is publicly available and can be accessed via the projects GitHub repository (https://github.com/OpenDevSci/LongDev-ABCD)
